# Health Promotion and Diagnosis of Oral Diseases in Institutionalized Elderly People: An Experience Report

**DOI:** 10.3390/ijerph22071097

**Published:** 2025-07-11

**Authors:** Isadora Lima Pereira, Fabio Augusto Ito, Ademar Takahama Júnior, Tiago Carvalho dos Santos, Paulo Sérgio da Silva Santos, Camila Lopes Cardoso, Heliton Gustavo de Lima

**Affiliations:** 1 Department of Oral Medicine and Pediatric Dentistry, State University of Londrina, Londrina 86057-970, Brazil; isadoradelimape@gmail.com (I.L.P.); fabioaito@gmail.com (F.A.I.); ademartjr@uel.br (A.T.J.); 2 Department of Surgery, Stomatology, Pathology and Radiology, Bauru School of Dentistry, University of São Paulo, São Paulo 17012-901, Brazil; tiagocsantos@usp.br (T.C.d.S.); paulosss@fob.usp.br (P.S.d.S.S.); cardosolopes@usp.br (C.L.C.); 3 Department of Stomatology, Federal University of Parana, Curitiba 80210-170, Brazil

**Keywords:** oral health, institutionalized elderly people, oral rehabilitation, oral diseases

## Abstract

This study presents the findings of an academic extension project focused on promoting oral health and diagnosing oral lesions in institutionalized elderly individuals. The project involved visits by students and faculty to two nursing homes in southern Brazil. Data collection included extraoral and intraoral clinical examinations and educational activities such as lectures and the distribution of printed materials on oral and denture hygiene. According to caregiving staff, oral hygiene, including denture cleaning, was generally performed once daily during morning showers. A total of 118 older adults (68 males and 50 females; mean age 76.1 ± 8.6 years) were examined. Forty-nine used dentures, of whom only 24 (49%) reported satisfaction with their prostheses. In total, 42 oral lesions were identified, mainly angular cheilitis (8), inflammatory fibrous hyperplasia (7), irritation fibroma (7), frictional hyperkeratosis (7), prosthetic stomatitis (5), actinic cheilitis (3), traumatic ulcers (3), and leukoplakia (2). Educational sessions also targeted caregivers, offering practical guidance for improving hygiene practices. The results underscore the need for better oral care and improved access to dental services for institutionalized elderly populations. Academic extension activities play a valuable role in health promotion and in training future professionals in elderly care.

## 1. Introduction

Increases in life expectancy and declines in mortality have resulted in considerable growth in the elderly population. This demographic shift poses significant challenges for health policies, as systemic health problems increase with age, adversely affecting oral health. Conversely, oral health problems can exacerbate systemic conditions, negatively impacting the overall health and quality of life of the elderly [1].

Common oral health problems in the elderly include mainly hyposalivation/xerostomia, opportunistic infections such as candidiasis, periodontal disease, caries, and oral manifestations of systemic diseases [2]. Hyposalivation has been demonstrated to adversely affect swallowing, communication, and contribute to halitosis, chronic parotitis, candidiasis, and dental caries [3]. The use of substandard prostheses can lead to various undesirable outcomes, including non-neoplastic proliferative processes, angular cheilitis, and traumatic ulcers [4]. The elderly population residing in care homes or institutions may face increased challenges in personal care, including oral care, which can adversely impact the quality of care and result in poor oral health outcomes [5].

The etiology of most oral alterations in the elderly is multifactorial, involving the individual’s care habits over their lifetime and the systemic conditions prevalent in this age group. Notably, systemic diseases not only contribute to oral alterations, but their treatments can also significantly impact oral health.

Local factors, both traumatic and non-traumatic, are commonly observed in this demographic due to the frequent need for oral rehabilitation. These factors include mechanical irritation (caused by habitual biting of the mucosa or irritation from ill-fitting prosthesis edges), thermal irritation (related to food intake or smoking), biofilm accumulation on natural and artificial surfaces, changes in salivary secretion, nicotine addiction, and alcohol abuse [6,7].

The oral health of the elderly, particularly those residing in care institutions, can also be influenced by functional capacity and self-care limitations. Therefore, raising awareness among healthcare professionals about the unique oral health challenges faced by institutionalized elderly populations is crucial. The identification of risk factors associated with poor oral health is imperative for implementing effective and targeted interventions [8].

Edentulism in old age is not an inevitable consequence of aging but rather the cumulative result of untreated oral diseases over time, including dental caries and periodontal diseases. These conditions arise not only from biological factors but also from socioeconomic and behavioral influences [9]. Tooth loss can result in difficulties in eating, reduced phonation capacity, and a range of detrimental effects on nutrition, esthetics, and psychological well-being, such as decreased self-esteem and social isolation [10,11,12].

The primary objectives of prostheses in oral rehabilitation are to restore masticatory function, esthetics, phonetics, and patient comfort. It is important to emphasize that tooth loss can lead to alterations affecting an individual’s emotional well-being. Therefore, oral health surveillance and promotion should be fundamental objectives for the multidisciplinary team responsible for elderly care, including dentists, dental hygienists, geriatricians, nurses, and caregivers. Promoting oral health is particularly crucial for ensuring the quality of life of the elderly [13].

Addressing oral hygiene in relation to removable prostheses is imperative, as evidence indicates that inadequate hygiene practices among prosthesis users can lead to specific oral and systemic infections [14]. The base of a prosthesis, typically made of acrylic resin, can be colonized by *Candida* spp. and bacteria from intra- and extra-oral sources. This colonization can result in conditions such as prosthetic stomatitis, which is highly prevalent among the elderly, as well as the presence of potential respiratory pathogens on prosthetic surfaces [15,16].

Recent studies have demonstrated the effectiveness of disinfection methods for prostheses when used in conjunction with antifungal therapy in the treatment of prosthetic stomatitis [17,18]. In addition to lesions caused by microorganisms accumulating on prosthetic surfaces, trauma from poor prosthesis adaptation to the alveolar ridge is also common [19]. During the physical examination, it is essential to be vigilant for malignant lesions, such as oral cancer, or potentially malignant lesions like leukoplakia and erythroplakia [20]. This population may also be exposed to other factors, such as tobacco, alcohol, and ultraviolet radiation, which are strongly associated with the development of oral cancer [21]. This represents a significant health threat to adults and the elderly in both high- and low-income countries. The disease manifests more severely in males than in females, and it is a particularly salient concern among individuals over 65 years old [13].

Currently, there is a lack of information for the elderly regarding the repercussions of systemic diseases on oral health and vice versa, as well as the effects of using multiple medications in the oral cavity. In this context, the importance of oral care and rehabilitation should be emphasized not only to the patient but also to the care team assisting them daily, including nursing technicians, caregivers, or companions. This focus can result in improved quality of life and early detection of potentially malignant lesions or oral cancer [19].

The objective of this manuscript is to present the findings of an extension project that sought to enhance the health status and diagnose oral lesions in institutionalized elderly individuals within two nursing facilities in a Brazilian city. The primary focus of this project was on the promotion of oral health through educational initiatives, with a secondary emphasis on the identification of potential oral lesions, both those associated with and those not associated with oral rehabilitation.

## 2. Materials and Methods

This study was developed as part of an oral pathology outreach project focused on institutionalized older adults. Initially, scientific meetings were held to train the undergraduate dental students involved in the project. The training addressed relevant topics such as aging biology, the impact of systemic conditions on oral health, the oral side effects of common medications in the elderly, oral lesions related or unrelated to prosthetic use, and oral hygiene practices. Printed educational materials were created, including illustrated guides titled “Cleaning dentures” and “Attention to oral diseases associated with denture use”.

Prior to clinical activities, a technical visit was conducted by the teachers to assess the physical and organizational structure of the long-term care facilities (LTCFs). This included evaluating staff workflows, daily routines of the elderly, and existing oral hygiene protocols. Two LTCFs participated in the study, both located in Londrina, Parana, and selected based on convenience and institutional approval.

Educational sessions were then delivered to both the institutionalized older adults and the caregiving staff. These sessions included lectures and hands-on demonstrations focused on oral and prosthetic hygiene, illustrated with clinical images of prevalent oral diseases caused by poor hygiene or ill-fitting dentures. The topics covered also included potentially malignant disorders and oral cancer, highlighting associated risk factors such as tobacco, alcohol, and ultraviolet exposure. A visual poster outlining step-by-step denture hygiene was designed and distributed to both institutions.

This poster detailed a practical protocol for daily and periodic denture care. It recommended cleaning the denture after meals and before bedtime using soft-bristled brushes and mild soap, while avoiding abrasive toothpaste that could damage the prosthesis. For additional disinfection, it advised immersing the dentures overnight every 15 days in either (1) a sodium hypochlorite solution (1 teacup of filtered water with 15 mL of 2–3% sodium hypochlorite, for complete dentures) or (2) a sodium bicarbonate solution (1 cup of filtered water with 1 teaspoon of bicarbonate, for full or partial dentures), followed by brushing under running water. The protocol also included the cleaning of the oral tissues—palate, cheeks, tongue, and edentulous ridges—using a soft toothbrush with toothpaste or a moistened cloth/gauze. Specific instructions were provided on removing denture adhesive residues, emphasizing gentle techniques such as rinsing with warm water, massaging the gums, and using tissue-wrapped fingers to aid in removal.

During clinical visits, a standardized clinical form was used to collect sociodemographic data, general health and medication history, habits (such as smoking and alcohol use), and prosthetic use information. Extraoral and intraoral examinations were performed on all participating older adults by trained examiners under the supervision of two board-certified oral and maxillofacial pathologists. A comprehensive extraoral physical examination was conducted to evaluate the presence of potential facial asymmetries, congenital anomalies, esthetic discolorations, and developmental alterations. The intraoral examination aimed to identify structural and functional abnormalities, with particular attention to the oral mucosa, salivary glands, tongue, and other relevant anatomical sites. This comprehensive assessment was designed to detect oral lesions potentially related or unrelated to the use of dental prostheses. Lesions were recorded following a systematic diagnostic protocol based on well-established clinicopathological criteria, under the direct supervision of two board-certified oral and maxillofacial pathologists. When deemed clinically necessary, incisional biopsies were performed, and histopathological analysis was conducted to confirm the diagnosis and enhance diagnostic accuracy. Furthermore, a detailed assessment of denture condition was carried out, including an evaluation of fit, the integrity of the acrylic base, the presence of biofilm, the condition of the dentures, and user-reported satisfaction.

As a reinforcement, each elderly person was given a review of oral hygiene and prosthetic instructions, as well as a reminder of important points about oral lesions. Additionally, the caregiving team received guidance to provide appropriate care for incapacitated elderly individuals. These strategies aimed to heighten awareness of the importance of oral health and to encourage consultation with professionals if any oral changes occur, especially those that persist beyond 15 days.

## 3. Results

A total of 118 elderly people were assessed, comprising 68 men and 50 women, with a mean age of 76.1 ± 8.6 years. Among them, 49 participants were denture wearers, but only 24 (49%) reported being satisfied with their dentures. Those experiencing ill-fitting or uncomfortable prostheses were referred to specialized dental services for the fabrication of new dentures. Additionally, dry mouth (xerostomia) was reported by 29 individuals, representing 24.6% of the study population. A total of 20 participants (16.9%) were identified as active smokers at the time of assessment.

Intraoral physical examination revealed that 33.9% of the participants presented some form of oral disease. A total of 42 oral lesions were identified, with the most common being angular cheilitis (8), inflammatory fibrous hyperplasia (7), irritation fibroma (7), frictional hyperkeratosis (7), prosthetic stomatitis (5), actinic cheilitis (3), traumatic ulcers (3), and leukoplakia (2) (Table 1). Elderly patients diagnosed with these conditions received appropriate treatment within the nursing home setting. Lesions requiring biopsy, such as leukoplakia, irritation fibroma, and inflammatory fibrous hyperplasia, were referred to the Dental Outpatient Clinic at the University Dental Clinic of the State University of Londrina.

The management of traumatic ulcers, denture stomatitis, and angular cheilitis involved adjustments to ill-fitting dentures and, when necessary, the fabrication of new prostheses. Participants were also instructed on appropriate denture hygiene, and topical antifungal therapy was prescribed in cases with suspected fungal involvement.

Regarding oral hygiene practices, although precise numerical data were not available, reports from the caregiving staff indicated that hygiene was typically performed once daily during morning showers. Among male residents, some were able to perform oral hygiene independently, whereas none of the female residents were reported to do so without assistance.

In addition to clinical care, the project promoted educational activities within nursing homes, including staff training sessions and knowledge exchange. It became evident that the caregiving teams had a limited understanding of elderly oral health and were following inadequate oral hygiene protocols. To address these gaps, the team provided guidance on geriatric oral health, denture care, and the recognition of common oral lesions. Practical demonstrations, supported by printed educational materials summarizing key recommendations and protocols, proved highly beneficial. These actions resulted in marked improvements in oral hygiene and prosthesis conditions among residents. The project was well received by both the professionals and the elderly individuals in both facilities.

## 4. Discussion

Aging brings changes that can significantly affect quality of life and autonomy. Functional limitations often compromise daily activities such as feeding and oral hygiene, requiring support from caregivers. These challenges are particularly evident in institutionalized elderly populations, such as those in nursing homes, where systemic diseases, dependency, and social isolation further compromise overall health [22,23]. Impairments in both instrumental and basic activities of daily living become more prevalent with advancing age, underscoring the need for supportive interventions to enhance the quality of life for the elderly [22,23].

The SB Brazil 2020 survey [24], which was extended due to the COVID-19 pandemic and concluded in the first half of 2024, provides updated insights into the oral health conditions of elderly Brazilians. The findings indicate that, although there has been a reduction in the number of decayed, missing, and filled teeth (DMFT) compared to 2010, a significant proportion of the elderly population—approximately 70%—still requires the use of dental prostheses [24]. This reflects a state of vulnerability in the oral health of the elderly and suggests a potential history of inadequate care, compounded by a care model historically marked by a high prevalence of tooth extractions [25].

Despite improvements proposed by the National Oral Health Policy, including prosthetic rehabilitation at the primary care level, the actual use of dentures remains insufficient, especially among individuals from lower socioeconomic backgrounds [24,25,26,27]. Studies show that elderly patients often use poorly fitting prostheses and face barriers to access adequate maintenance or replacement [28,29]. This may stem from both the insufficient structuring of the public system and the elderly’s reluctance to acknowledge their deteriorating oral health [28,29].

Among the elderly assisted, only 49% of denture wearers reported satisfaction with their prostheses, which is consistent with previous Brazilian studies highlighting the widespread use of unsatisfactory dental prostheses among the elderly [30,31]. Additionally, 33.9% of individuals presented oral lesions, with angular cheilitis, hyperplastic lesions, traumatic ulcers, and prosthetic stomatitis being the most common findings. Similar data were observed in studies carried out in long-term care institutions, where poor denture hygiene, ill-fitting prostheses, and the loss of the vertical dimension of occlusion were key contributing factors [31,32].

Oral lesions associated with denture use include denture stomatitis, angular cheilitis, inflammatory fibrous hyperplasia, and traumatic ulcers [32]. While denture stomatitis and angular cheilitis are frequently linked to Candida albicans colonization, particularly due to its ability to adhere to acrylic surfaces and form biofilms, inflammatory fibrous hyperplasia and traumatic ulcers are primarily mechanical in origin [32,33]. Nevertheless, poor prosthetic retention, reduced salivary flow, and loss of vertical dimension can predispose to or exacerbate all these conditions.

Dry mouth (xerostomia) was reported by 24.6% of the elderly individuals assisted through our extension project, aligning with findings by Huang et al. [34], who identified dry mouth as a prevalent self-perceived condition among institutionalized elderly, particularly related to denture use and a lack of regular oral examinations. Reduced salivary flow compromises oral defense mechanisms, predisposing to candidiasis, mucosal injuries, and reduced denture retention and comfort [32].

The development of traumatic ulcers has been related to fractures in the denture base, instability, irregular repairs, overextension, and occlusal imbalance [31]. Angular cheilitis is often associated with vertical dimension loss, chronic candidiasis, vitamin B deficiencies, and anemia. In edentulous patients, the collapse of the lower third of the face may favor salivary accumulation in labial commissures, fostering Candida colonization [31]. Poor hygiene practices and a lack of caregiver training are significant public health issues that impact oral and systemic health.

Continuous denture wear, especially during sleep, has been associated with a higher risk of aspiration pneumonia and impaired local circulation, reinforcing the link between oral and systemic health [35]. Moreover, denture deterioration over time—due to wear, cracks, or a loss of vertical dimension—can increase the risk of oral trauma and mucosal lesions. Still, elderly individuals frequently resist replacing their prostheses, despite clinical indications for doing so within two to five years of use [36,37,38].

Within the context of our extension project focused on health promotion and education, 16.9% of participants reported smoking habits. Smoking is a well-established risk factor for several oral diseases, including leukoplakia and oral cancer. This finding underscores the importance of targeted educational strategies and tobacco cessation initiatives aimed at this population [39,40,41].

Furthermore, potentially malignant lesions, such as leukoplakia, though not common in our sample, reinforce the importance of routine oral examination and early referral for specialized care. Some studies emphasize the challenges of diagnosing such conditions in institutionalized populations due to infrequent dental visits and limited access to specialized services [42,43,44]. Our strategy of referring suspected cases to a university-affiliated dental clinic ensured proper diagnosis and illustrates the importance of academic engagement in mitigating care gaps.

Another important aspect of this study was the observation of hygiene practices within institutions. Caregivers often reported performing oral hygiene care, but inconsistencies in technique and regularity were observed. These observations are consistent with the literature, which highlights the need for proper caregiver training to ensure effective hygiene routines [45,46].

Denture deterioration due to factors such as wear and tear, uneven edges, and cracks in the teeth or acrylic contributes to an increased risk of oral injuries. Elderly patients often exhibit reluctance to replace their prostheses; however, after two to five years, the deterioration of the artificial teeth results in a substantial loss of the vertical dimension of occlusion, necessitating a re-evaluation for potential replacement [36]. This condition leads to increased mucosal sensitivity, diminished retention, and the development of mucosal lesions.

Maintaining natural dentition or rehabilitating it with dental prostheses improves health outcomes and quality of life but requires proper hygiene and routine monitoring [38]. Denture wearers should be educated on correct hygiene techniques, including daily brushing, nighttime removal, and chemical cleaning at least biweekly. Dental evaluations should occur regularly to detect mucosal changes or the need for prosthesis adjustment or replacement [31,38]. These practices should be part of public health strategies promoting aging with dignity and autonomy.

A relevant condition observed in this age group, especially among males, is oral cancer. Elderly individuals should be assessed and educated on self-examination methods and the risk factors associated with malignancy, such as tobacco, alcohol, and sun exposure [39]. Early diagnosis significantly improves prognosis and survival, particularly considering the asymptomatic nature and challenging anatomical locations commonly affected by oral squamous cell carcinoma [40].

Despite the absence of quantitative metrics in this extension project, our experience has had a significant impact on the institutionalized elderly, caregivers, and students involved. The initiative helped demystify oral health issues, empowered caregivers with knowledge, and exposed students to the realities of long-term care settings. Through dialog and action, the project successfully created a bridge linking academia to the community, promoting collective responsibility in addressing the oral health of vulnerable populations.

## 5. Conclusions

The academic extension activities detailed in this report have heightened awareness among elderly individuals and their caregivers regarding the importance of oral health. These initiatives have led to positive behavioral changes and the establishment of effective protocols for oral hygiene and denture care, thereby enhancing the health and well-being of the elderly population. Notably, elderly individuals presenting with oral lesions or unsatisfactory dentures were referred to specialized dental services for appropriate treatment. Furthermore, by engaging directly with this demographic, participating teachers and students gained comprehensive theoretical and practical knowledge in geriatric dentistry, contributing to their professional development.

## Figures and Tables

**Table 1:** Oral diseases identified during intraoral examination of institutionalized elderly individuals.

Oral Disease	Elderly (*n*)
Angular cheilitis	8
Inflammatory fibrous hyperplasia	7
Irritation fibroma	7
Frictional hyperkeratosis	7
Prosthetic stomatitis	5
Traumatic ulcer	3
Actinic cheilitis	3
Leukoplakia	2
**Total**	**42**

## Data Availability

The original contributions presented in this study are included in the article. Further inquiries can be directed to the corresponding author.
